# Association between penile ischemia duration and complications after hypospadias repair in children

**DOI:** 10.3389/fped.2026.1763150

**Published:** 2026-03-13

**Authors:** Malin Dönig, Volker Sander, Ralf-Bodo Tröbs, Jochen Hubertus, Matthias Nissen

**Affiliations:** 1 Department of Pediatric Surgery, Marien Hospital Witten, Ruhr-University Bochum, Witten, Germany; 2 Department of Pediatrics, Gemeinschaftskrankenhaus Herdecke, Herdecke, Germany; 3 Department of General, Visceral and Pediatric Surgery, St. Vincenz Krankenhaus, Paderborn, Germany

**Keywords:** hypospadias repair, ischemia duration, meatal stenosis, penile tourniquet, postoperative complications, urethral fistula

## Abstract

**Introduction:**

Clinical evidence regarding the impact of intraoperative penile ischemia during pediatric hypospadias repair remains inconclusive. Although experimental studies suggest that ischemia–reperfusion injury may impair wound healing, clinical data on the relationship between ischemia duration and postoperative outcomes in children are limited. This study aimed to determine whether the duration of intraoperative penile ischemia is associated with postoperative complications—specifically urethral fistula formation and meatal stenosis—in patients undergoing primary hypospadias repair.

**Methods:**

We conducted a retrospective, single-center cohort study of pediatric patients who underwent primary hypospadias repair with intraoperative penile tourniquet application over a 12-year period. The primary outcome was the occurrence of postoperative complications, specifically meatal stenosis or urethral fistula, requiring revision surgery. Secondary analyses assessed tourniquet duration, patient demographics, and key perioperative variables to identify potential risk factors for adverse outcomes.

**Results:**

Ninety-four patients were included. Postoperative complications requiring redo surgery occurred in 17 patients (18%), comprising urethral fistula (*n* = 12), meatal stenosis (*n* = 3), or both (*n* = 2). Median ischemia duration did not differ significantly between patients with and without complications (39 [34–45] vs. 35 [30–42] min; *p* = 0.15). No significant differences were observed in operative time, biometric variables, hypospadias severity, suture material, stenting duration, or postoperative hospital stay. Mean follow-up was 76 months. A structured literature review revealed that ischemia duration is inconsistently reported across studies, limiting comparability.

**Conclusions:**

Intraoperative penile ischemia duration within the mid-range commonly applied in clinical practice was not associated with an increased risk of postoperative fistula or meatal stenosis following primary hypospadias repair. These findings support the safety of standard penile tourniquet use during routine surgical procedures. However, prospective studies with standardized documentation of ischemia duration are needed to establish evidence-based safety thresholds and to further clarify potential ischemia–reperfusion effects.

## 1 Introduction

Hypospadias is one of the most common paediatric urological malformations, affecting approximately 1 in 200–300 live male births (1–4). This congenital anomaly of the urogenital tract results from intrauterine androgen deficiency, particularly after the 14th week of gestation, leading to the incomplete fusion of the urethral folds (5). The aetiology of hypospadias is considered multifactorial, with both genetic predispositions and environmental factors contributing to its development (6). Postoperative complications, including urethral fistula and meatal stenosis, remain common, with approximately half occurring within the first year after corrective surgery (7). Various biometric, intraoperative, and perioperative factors have been implicated as potential contributors to these complications (8–10). The severity of hypospadias correlates with the complexity of repair; more proximal defects require extensive reconstruction and are associated with higher complication rates (11). Intraoperative bleeding management is critical during hypospadias repair, as excessive blood loss can affect surgical precision and postoperative outcomes (12). The application of a penile tourniquet is widely used to reduce blood loss and improve surgical visibility (13). However, tourniquet use can alter coagulation parameters (14), and prolonged application may induce ischaemia-reperfusion injury (12, 15). Despite these considerations, the association between tourniquet duration and postoperative complications has not been systematically evaluated. This retrospective study assessed the relationship between intraoperative penile ischemia duration and the occurrence of urethral fistula or meatal stenosis. The findings provide clinically relevant insights into the safety profile and limitations of tourniquet use in pediatric hypospadias repair.

## 2 Patients and methods

This retrospective, single-centre study included 433 consecutive patients diagnosed with hypospadias (ICD-10: Q54) who underwent primary surgical repair between 2010 and 2021. The duration of tourniquet application, defined by the length of continuous or cumulative occlusion, was documented in 121 cases. Continuous ischemia is defined as uninterrupted tourniquet application. Cumulative ischemia is defined as the sum of all tourniquet occlusion periods within a single procedure and could be reliably determined from the operative records. Intervals of tourniquet release were explicitly excluded from the cumulative ischemia duration. The release was not standardised and was performed at the discretion of the operating surgeon. As a result, detailed information on release frequency and duration could not be reliably reconstructed from the operative records. As a result, detailed information on release frequency and duration could not be reliably reconstructed from the operative records. Patients with only nonspecific documentation (e.g., “below 30 min”) were excluded. Additional exclusion criteria were concomitant penile epinephrine application (*n* = 15), simultaneous surgery at another site (*n* = 5), presence of a metabolic disorder (*n* = 1), or 3° hypospadias (*n* = 6) Patients with metabolic disorders were excluded to minimize potential systemic confounding, as such conditions may independently impair wound healing and tolerance to ischemia. Grade III hypospadias cases were excluded because they typically require more complex and often multi-stage reconstructive approaches with substantially different operative duration, tourniquet application patterns, and baseline complication risks, which would confound analyses focused on primary single-stage repairs. Noncomplicated cases comprised postoperative findings not necessitating reoperation, including epidermal cysts (*n* = 2), oedema (*n* = 2), catheter dislocation (*n* = 2), and preputial skin excess (*n* = 3). To simplify the causal attribution of tissue injury, these cases were not regarded as tourniquet-associated, resulting in a final cohort of 94 patients for analysis. The surgeries were performed by two consultant-level surgeons at a tertiary pediatric surgery department. Clinical notes, operative reports, and follow-up examinations provided data. The principal outcome measure was the duration of intraoperative penile ischaemia, examined as a function of the postoperative course, distinguishing between cases complicated by meatal stenosis or urethral fistula requiring secondary intervention and those with an uneventful postoperative outcome. Postoperative complications were identified based on clinical documentation in operative reports and follow-up records and were defined as urethral fistula or meatal stenosis requiring surgical revision; standardized imaging or functional diagnostic criteria were not uniformly applied due to the retrospective study design. Secondary endpoints included a systematic assessment of the association between tourniquet duration and relevant clinical covariates, including patient demographics, hypospadias severity, and operative parameters such as suture characteristics, operative time, and postoperative length of stay. Hemostasis was achieved using a circular, non-pneumatic Penrose tourniquet applied at the base of the penis, with cumulative tourniquet duration recorded for each procedure. The tourniquet was manually tightened by the operating surgeon until adequate hemostasis was visually confirmed, indicated by skin blanching and venous collapse. All patients received prophylactic intravenous antibiotics. Hypospadias severity was classified according to Smith et al. (16) as glandular (grade I), coronal/subcoronal/distal/midshaft (grade II), or proximal penile/penoscrotal/scrotal/perineal (3°). Penile chordee was assessed intraoperatively via artificial saline erection; if present, correction was performed during hypospadias repair. Snodgrass (tubularized incised plate) procedure: The urethral plate was incised along the midline with lateral paraurethral incisions, glans wings were mobilised, and urethroplasty was performed with 7/0 or 8/0 interrupted suture, reinforced by a running suture. Mathieu procedure: A distal meatal-based flap was raised, tubularized, and rotated to form the neourethra, with glans wings approximated to create a slit-like neomeatus at the glans tip. Postoperative urinary diversion was achieved either by transurethral catheterization alone or by combined transurethral and suprapubic catheterization, according to institutional practice. In most cases, a suprapubic catheter was inserted during hypospadias repair and subsequently removed once spontaneous voiding through the neourethra was successfully established. A chimney dressing with silicone mesh was used as the standard postoperative dressing method.

Data were collected using Excel 2010 (Microsoft, Redmond, WA, USA) and analysed using OriginPro 2021 (OriginLab, Northampton, MA, USA). The normality of continuous variables was assessed using the Kolmogorov–Smirnov test at a significance level of 0.05. For variables that did not meet normality assumptions, no data transformation was applied in order to preserve the original clinical scale of measurement. Instead, non-parametric statistical methods were chosen. Non-normally distributed data are described as medians with first (Q1) and third (Q3) quartiles, unless stated otherwise. Comparisons between groups were performed using the Mann–Whitney U-test. Categorical variables were summarised as frequencies and percentages and analysed using the two-tailed Fisher's exact test, where appropriate. Statistical significance was defined as *p* ≤ 0.05. The study cohort consisted of all consecutive eligible patients treated during the study period; no formal *a priori* sample size calculation was performed due to the retrospective observational study design.

## 3 Results

The basic biometric and procedural characteristics of the 94 patients included are summarised in Table 1. Patients were dichotomised into those with complicated (*n* = 17) and noncomplicated (*n* = 77) postoperative courses. Complications included urethral fistula (*n* = 12), meatal stenosis (*n* = 3), or both (*n* = 2), each requiring redo surgery.

**Table 1:** Biometric and procedural characteristics of the patients.

Variable	Complicative	Non-complicative	*p*
*n*	17	77	
Age (months)	19 (11–36)	17 (11–35)	0.84
Weight (kg)	13 (9–18)	12 (10–15)	0.67
Gestational age (wks)	38 (37–40) (*n* = 16)	38 (37–40) (*n* = 43)	0.64
Birth weight (kg)	3.3 (3.0–3.7) (*n* = 13)	3.3 (2.9–3.7) (*n* = 50)	1
Length of stay(d)	7 (6–9)	7 (5–8)	0.10
Operative time (min)	91 (80–110)	80 (70–100)	0.17
Cumulative ischemia duration (min)	39 (34–45)	35 (30–42)	0.15
Tourniquet (continuous/ interval; *n*)	16/2	75/2	0.16
Follow-up (months)	87 (65–101)	76 (68–90)	0.43
Hypospadias grading			0.48
1°(*n*)	4	12	
2°(*n*)	13	65	
Associated chordee (y/n)	3/14	22/55	0.39
Type of repair			0.75
Snodgrass	7	24	
Mathieu	8	44	
others	2	9	
Suture material^a^			0.82
Vicryl	9	47	
Safil	8	30	
PDS (additional)	15	65	
Transurethral stenting duration (days)	7 (6–8)	6 (5–7)	0.27
Stenting method			0.78
Urethral catheter alone	5	27	
Urethral and suprapubic catheter	12	50	

a Size 7/0 or 8/0; n.s. not stated; PDS *Polydioxanone.*

Baseline biometric characteristics, including age, gestational age, weight, and birth weight, were comparable between the groups. Overall operative time and cumulative ischemia duration did not differ significantly. Continuous tourniquet application was predominantly employed in both cohorts, and the duration of postoperative follow-up was similar. Revision-requiring complications occurred predominantly early after surgery, with a median time to revision of 9 (range 0–47) months. Second-degree hypospadias was the most frequent anatomical subtype, and the Mathieu procedure was the most commonly performed technique. No significant intergroup differences were observed with respect to suture material, stenting duration, or stent type.

## 4 Discussion

This retrospective study aimed to assess the influence of tourniquet duration during hypospadias repair on postoperative outcomes. The analysis demonstrated that there was no significant variation in the tourniquet duration between patients who encountered complications, such as fistula or stenosis requiring revision surgery, and those who experienced an uncomplicated recovery. Additionally, the study found no significant differences in operative time or hospital stay length between the two groups.

To the best of our knowledge, the tourniquet duration has not been previously examined as a primary factor affecting the postoperative development of meatal stenosis or urethral fistula in pediatric patients who underwent primary hypospadias repair.

Current research primarily focuses on evaluating the safety of various methods and their postoperative outcomes rather than directly investigating the impact of ischaemia. A review of the literature reveals a lack of clinical studies that systematically document and analyse tourniquet duration as an independent risk factor for postoperative complications following hypospadias surgery. Table 2 summarizes a literature review on the use of tourniquets in hypospadias repair, highlighting considerable variability in study design, sample size, severity of hypospadias, surgical techniques, and primary endpoints, which constrains the comparability of results.

**Table 2:** Literature data on the outcomes of pediatric hypospadias repair utilizing a penile tourniquet.

Reference	Bleeding control	n	Study	Overall complications (%)	Fistula (%)	Stenosis (%)	Fistula & stenosis (%)	Complications other than stenosis/fistula (n)	Hypospadias grading (°)	Mode of repair (n)	Tourniquet duration (min)	Operative time (min)	Length of stay (d)	Follow up (m)
This study.	T	94	r	17 (18)	14 (15)	5 (5)	2 (2)	None	1–2	SR, M, others	C: 39 (34–45) Non-C: 35 (30–42)	C: 91 (80–110) Non-C: 80 (70–100)	7	C: 87; Non-C: 76
Aminsharifi et al. (17)	T	40	p	5 (13)	2 (5)	5 (13)	2 (5)	none	1–2	SR, M-IP	R20	S: 95 M-IP: 100	2	12
Belman (18)	T & EC	75	r	9 (12)	4 (5)	3 (4)	0	megaurethra (1), disruption (1)	2–3	OSR, TIOU, TIPT	R10	n.s.	<1	n.s.
Cloutier (19)	T	22	p	5 (23)	5 (23)	0	0	none	n.s.	DBR	n.s.	n.s.	n.s.	n.s.
Alizadeh et al. (20)	T E	70 [T:35;E:35]	p	18 (26) [T:9 (26); E:10 (29)]	2 (3) [T: 0; E: 2 (6)]	6 (9) [T: 2 (6) E: 4 (11)]	0	edema (1), hematoma (1), bleeding (1), infection (1), necrosis (1), dehiscence (5)	1–3	SR	½ OT	T: 65,3 E: 70,12	n.s.	9
Kassem et al. (21)	T E & EC	60 [T:30; E:30]	p	31 (52) [T: 19 (63); E: 12 (40)]	5 (8) [T: 2 (7); E: 3 (10)]	3 (5) [T: 1 (3) E: 2 (7)]	0	urethral stricture (3), hematoma (6), edema (10), bleeding (3), infection (1)	1–2	SR	≤40/R40	T: 76,6 E: 88,5	n.s.	n.s.
Günendi and Kocaman (22)	T & EC DE & EC	78	r	12 (15) [T: 2 (5); E: 10 (26)]	4 (5) [T: 1 (3), E: 3 (8)]	6 (8) [T: 1 (3) E: 5 (13)]	0	dehiscence (1), urethral diverticulum (1)	1–3	SR	R10	n.s.	n.s.	6
Helmy et al. (23)	T & EC Non-T & EC	102 [T:49; Non-T:53]	p	28 (27) [T: 16 (33) Non-T: 12 (23)]	7 (7) [T: 5 (10) Non-T: 2 (4)]	11 (11) [T: 7 (14) Non-T: 4 (8)}	0	Infection (5), dehiscence (5)	1	SR	≤30/R30	T: 54,7 Non-T: 60,8	n.s.	12
Taicher et al. (24)	T E	395	r	22 (6)^a^	n.s.	n.s.		dehiscence (n.s.)	1–3	n.s.	n.s.	139,7	n.s.	n.s.
Koca et al. (25)	T T&EC	59 [T:32;T&EC:27]	p	9 (15)	6 (10) [T:3 (9); T&B:3 (11)]	3 (5) [T: 2 (6) T & B: 1 (4)]	0	none	1	SR	≤20/R20	T: 76,8 T&B: 82,6	2	n.s.
Redman (26)	T	146	r	1 (1)	0	0	0	bleeding (1)	n.s.	n.s.	28 (12–50)	n.s.	n.s.	n.s.
Herrera et al. (27)	T	147	r	9 (6)	5 (3)	3 (2)		skin tag (1)	1–2	SR	n.s.	67	n.s.	18,4
Akbiyik et al. (28)	T	496	r	48 (10)	25 (5)	27 (5)	4 (1)	dehiscence (1)	1–2	SR	n.s.	n.s.	10	24
Snodgrass et al. (29)	T	551	r	19 (4)	9 (2)	9 (2)	0	dehiscence (1)	1	SR	n.s.	N.s.	n.s.	8
Khan et al. (30)	T	428	r	320 (75)	166 (39)	24 (6)	n.s.	edema (122), bleeding (18), infection (19), dehiscence (19)	1–3	SR, TSB	n.s.	≤60, > 60	3	n.s.
Storm et al. (31)	T	422	r	103 (25)	43 (10)	11 (3)	n.s.	dehiscence (14), cicatrix (4), inclusion cyst (2), breakdown (1)	1–3	SR, TD	n.s.	108	n.s.	9
Nicolle et al. (32)	T	100	r	11 (11)	5 (5)	1 (1)	0	Haematoma (1), wound infection (1), displaced catheter (3)	1–3	B/C	n.s.	n.s.	n.s.	n.s.

T, tourniquet; E, epinephrine injection; EC, electrocautery; SR, Snodgrass repair; M-IP, Mathieu Incised Plate procedure; OT, operative time; R*x*, tourniquet released every *x* min; ≤ *x*/R*x*, tourniquet applied for a maximum of *x* min continuously or (/) released every *x* min if longer procedures were required; OSR, Onlay Split Repairs; TIOU, Transverse Island Onlay Urethroplasty; TIPT, Transverse Island Pedicle Tube; n.s., not stated; DBR, Denis Browne repair; TSB, Two-stage (Bracka); TD, Thiersch-Duplay; B/C, Byars-Cloutier modified technique.

a Allocation into T or E not further specified.

In hypospadias surgery, the severity of hypospadias influences the surgical approach, duration, and postoperative outcomes (11, 31). Distal forms are typically repaired in a single stage using standardized techniques like tubularized incised plate (TIP) urethroplasty, showing favourable outcomes (11). More severe hypospadias cases often require staged procedures and show higher complication rates. Our findings regarding the incidence of fistulas and stenosis are consistent with existing studies on distal and midshaft hypospadias (17, 21, 23, 27, 28). Our data revealed a similar overall complication profile, with a stenosis rate of 5.3%, but a higher incidence of fistula at 14.8%. The severity of hypospadias, particularly when complications occur, remains an area of uncertainty, especially concerning whether the duration of ischemia is a contributing factor.

All studies referenced utilized tourniquets as a method for haemorrhage control. An analysis of the data concerning tourniquet duration indicates that certain studies did not collect data on this aspect (19, 24, 27–31). The reported tourniquet duration does not consistently correlate with an increased complication rate. Comparably short tourniquet durations of 10 to 20 min lead to overall complication rates between 12% to 15% (17, 18, 22, 25). In contrast, longer tourniquet durations of up to 50 min (mean 28 min), as in the study by Redman et al. (26), show an exceptionally low overall complication rate of 0.7%, with no occurrences of fistulas or stenoses. The data presented by Khan et al. (30) is notable for its extended tourniquet duration and elevated complication rate, aligning with our hypothesis. In this retrospective cohort study involving 428 patients, tourniquet durations frequently surpassed 60 min, resulting in an overall complication rate of 74.8%, with 38.8% of cases involving fistulas and 5.6% involving stenoses. The discrepancy with Khan et al. is likely explained by differences in case mix, surgical complexity, and ischemia exposure. Their cohort included a high proportion of proximal hypospadias and two-stage repairs with frequently prolonged ischemia (>60 min), whereas our study focused on primary single-stage grade I–II repairs with ischemia clustered in the mid-range of routine practice. These differences suggest effect modification by severity and procedure duration rather than a universal independent ischemia effect. Nevertheless, these studies did not systematically investigate complications in relation to the tourniquet duration, thereby preventing any definitive conclusions regarding whether patients with prolonged tourniquet durations are more susceptible to complications. In the present study, the median tourniquet duration in the non-complicative group was 35 min, compared to 39 min in the complicative group, which is consistent with the mid-range of values reported in the literature. Statistically, there was no significant difference between extended tourniquet duration and the prevalence of complications. In a categorical analysis of tourniquet durations, complications necessitating revision were observed exclusively with durations ≥30 min: 0/5 (0–19 min group) and 0/9 (20–29 min group) compared to 9/43 (20.9%) in the 30–39 min group and 8/37 (21.6%) in the ≥40 min group. Consequently, dichotomizing durations into <30 vs. ≥30 min groups resulted in 0/14 vs. 17/80 (21.3%) complications, a difference that did not reach statistical significance (two-sided Fisher's exact test *p* = 0.07). The complication rates were comparable between the 30–39 and ≥40 min groups (20.9% vs. 21.6%; Fisher's exact *p* = 1.0), and a secondary 40-min cut-off (<40 vs. ≥40 min) was also non-significant (15.8% vs. 21.6%; Fisher's exact *p* = 0.585), noting that the <40 min group includes the event-free <30 min cases. These findings should be interpreted with caution, given the small sample sizes in the short-duration strata and the retrospective nature of tourniquet documentation, including unmeasured tourniquet pressure. Currently, there are no universally accepted guidelines concerning the maximum permissible duration for the use of a penile tourniquet (33). A tourniquet duration of 50 min is generally regarded as safe (26). Although local complications such as nerve injury, muscle damage, or skin trauma, substantial evidence from various medical disciplines—including cardiology, neurology, and transplant medicine—indicates that prolonged ischemia followed by reperfusion can lead to significant tissue damage, primarily mediated by oxygen-derived free radicals (34, 35).

Preclinical animal studies have explored the impact of ischemia-reperfusion on penile tissue. In a study conducted by Boskurt et al. (36) using a rabbit model, it was demonstrated that ischemia can adversely affect functional vascular damage after an ischemia duration of over 20 min. Kaya et al. showed that the application of a tourniquet for 30 min resulted in decreased levels of vascular endothelial growth factor (VEGF) and vascular endothelial growth factor receptor (VEGFR), suggesting impaired angiogenic activity. The study by Boybeyi-Turer et al. (37) provides compromised wound healing, increased endothelial injury markers [endothelial nitric oxide synthase (eNOS), intercellular adhesion molecule-1 (ICAM-1), e-selectin], and inhibited urothelial cell proliferation following penile ischemia of 10 min. Cakmak et al. (15) examined the effects of tourniquet application vs. epinephrine injection using a rabbit model. Histological changes in skin flaps and malondialdehyde levels were assessed as indicators of tissue damage. An ischemia duration of 10 min resulted in changes associated with reperfusion injury. Another study by Kajbafzadeh et al. (38), also conducted on rabbits, demonstrated that urothelial damage and apoptosis in smooth muscle could occur after approximately 30 min of ischemia. Importantly, these experimental findings predominantly describe molecular, endothelial, or histological alterations, which may remain subclinical and do not necessarily translate into revision-requiring complications such as urethral fistula or meatal stenosis, potentially explaining the lack of a significant association within the 30–45 min ischemia range observed in our clinical cohort.

Limitations of this study comprise the retrospective, single-center design and its small sample size. Moreover, we did not investigate ischemia effects on the molecular or histological level. This study has several limitations, including its retrospective, single-center design and relatively small sample size. In addition, ischemia-related effects were not assessed at the molecular or histological level. Because of the retrospective nature of the study, detailed reconstruction of reperfusion patterns—such as the timing and duration of intermittent tourniquet release—was not consistently feasible from operative records. Similarly, the precise length of urethral defects, which may influence postoperative outcomes, could not be reliably determined. Ischemic burden depends on both duration and applied pressure. In this study, a non-pneumatic Penrose tourniquet was used, which does not permit manometric standardization; therefore, tourniquet pressure could neither be measured nor reconstructed. Penrose tourniquets are known to exert variable and potentially high pressures (39–41), and well-defined pressure thresholds for penile applications remain limited (42). Future research should integrate time with devices capable of standardized pressure measurement or employ thin sensors. Postoperative urinary drainage is frequently analyzed in the literature as a factor influencing complications following hypospadias repair. However, strategies vary considerably between centers, complicating comparability. In our cohort, urinary diversion was achieved either through transurethral catheterization alone or in combination with suprapubic catheterization. According to the EAU Paediatric Urology guideline, in the context of distal hypospadias repair, suprapubic diversion is considered a viable option alongside transurethral drainage (e.g., dripping stent) or the absence of drainage (43). However, comparative clinical studies have reported favorable outcomes with suprapubic diversion compared to urethral stenting in one-stage repairs (44). Furthermore, a prospective randomized trial of distal TIP repairs indicated that suprapubic diversion can reduce the risk of fistula formation and that the addition of a small anterior urethral catheter may decrease the incidence of meatal stenosis and bladder spasm (45). Finally, minor complications were not included in the primary endpoint and may therefore be underestimated in retrospective documentation. While the present data provide valuable preliminary clinical insight, the findings should be interpreted with caution given the retrospective single-center design, limited sample size, absence of standardized tourniquet pressure measurements, and the inability to reconstruct detailed reperfusion patterns. These factors restrict the strength of causal inference and limit generalizability. Accordingly, the results should be considered hypothesis-generating rather than confirmatory, highlighting the need for prospective, standardized studies to further define safety thresholds for tourniquet application in pediatric hypospadias repair.

Further research should focus on examining damage secondary to tourniquet application on the molecular or cellular level. Penile lactate concentrations may serve as an indicator of tissue ischemia during surgical procedures. However, technical challenges may arise due to the limited volume of blood samples obtainable from the surgical field.

## 5 Conclusion

In conclusion, tourniquet duration within the observed range was not significantly associated with increased rates of fistula or meatal stenosis following primary hypospadias repair. While clinically meaningful, these findings should be interpreted cautiously given the retrospective nature of the study, limited cohort size, and lack of tourniquet pressure standardization. Rather than confirming safety thresholds, they should be regarded as hypothesis-generating, underscoring the need for future prospective studies incorporating standardized pressure measurement and perfusion monitoring.

## Data Availability

The raw data supporting the conclusions of this article will be made available by the authors, without undue reservation.
